# CONSISE statement on the reporting of Seroepidemiologic Studies for influenza (ROSES‐I statement): an extension of the STROBE statement

**DOI:** 10.1111/irv.12411

**Published:** 2016-08-09

**Authors:** Peter W. Horby, Karen L. Laurie, Benjamin J. Cowling, Othmar G. Engelhardt, Katharine Sturm‐Ramirez, Jose L. Sanchez, Jacqueline M. Katz, Timothy M. Uyeki, John Wood, Maria D. Van Kerkhove, the CONSISE Steering Committee

**Affiliations:** ^1^ Nuffield Department of MedicineCentre for Tropical Medicine and Global HealthUniversity of OxfordOxfordUK; ^2^ WHO Collaborating Centre for Reference and Research on Influenzaat the Peter Doherty Institute for Infectious DiseasesMelbourneAustralia; ^3^ WHO Collaborating Centre for Infectious Disease Epidemiology and ControlSchool of Public HealthLi Ka Shing Faculty of MedicineThe University of Hong KongHong Kong Special Administrative RegionChina; ^4^ National Institute for Biological Standards and ControlMedicines and Healthcare products Regulatory AgencyPotters BarUK; ^5^ Influenza Division, National Center for Immunization and Respiratory DiseasesCenters for Disease Control and PreventionAtlantaGAUSA; ^6^ Armed Forces Health Surveillance Center (AFHSC) and Cherokee Nation Technology Solutions, IncSilver SpringMDUSA; ^7^ Center for Global HealthInstitut PasteurParisFrance

## Abstract

**Background:**

Population‐based serologic studies are a vital tool for understanding the epidemiology of influenza and other respiratory viruses, including the early assessment of the transmissibility and severity of the 2009 influenza pandemic, and Middle East respiratory syndrome coronavirus. However, interpretation of the results of serologic studies has been hampered by the diversity of approaches and the lack of standardized methods and reporting.

**Objective:**

The objective of the CONSISE ROSES *‐*I statement was to improve the quality and transparency of reporting of influenza seroepidemiologic studies and facilitate the assessment of the validity and generalizability of published results.

**Methods:**

The ROSES‐I statement was developed as an expert consensus of the CONSISE epidemiology and laboratory working groups. The recommendations are presented in the familiar format of a reporting guideline. Because seroepidemiologic studies are a specific type of observational epidemiology study, the ROSES‐I statement is built upon the STROBE guidelines. As such, the ROSES‐I statement should be seen as an extension of the STROBE guidelines.

**Results:**

The ROSES *‐*I statement presents 42 items that can be used as a checklist of the information that should be included in the results of published seroepidemiologic studies, and which can also serve as a guide to the items that need to be considered during study design and implementation.

**Conclusions:**

We hope that the ROSES‐I statement will contribute to improving the quality of reporting of seroepidemiologic studies.

## 1 Background

For all pathogens, cases of symptomatic illness from infections that are detected through routine healthcare statistics, laboratory networks, or other surveillance and reporting systems are only a small proportion of infections affecting a population. Relying on case incidence detected by this information alone can lead to large underestimates of infection rates and overestimates of severity of illness among the general population.^1^ Prior to, or during the early stages of epidemics, serologic testing of affected populations can provide valuable information on the proportion of the population that have a low titer of cross‐reactive antibodies and may be at higher risk of infection with pathogens for which the primary determinant of protection is humoral immunity, for example, influenza viruses. During later stages of epidemics, or following epidemics, serologic testing can permit estimation of the number of infections that have occurred among the general population, which is important for determining important epidemiologic parameters, including the pathogen's transmissibility, the proportion of the population that remains susceptible to infection in subsequent epidemics, and the risk of severe disease or death conditional on infection. In the last decade, over 8000 studies have been published that are indexed with the Medical Subject Heading (MeSH) term “Seroepidemiologic Studies.” Approximately 500 studies of these are indexed with MeSH terms “Seroepidemiologic Studies” AND “influenza.”

The 2009 H1N1 influenza pandemic is an excellent example of how a variety of seroepidemiologic studies provided vital information to supplement what was available from clinical and laboratory surveillance data.^2^, ^3^, ^4^ Severity of the 2009 H1N1 pandemic was initially overestimated from the reports of high risks of severe disease among critically ill patients in adult intensive care units in Mexico City and Winnipeg.^5^, ^6^ However, inconsistencies in the reporting and standardization of both survey and laboratory methods have limited the comparability of results of 2009 H1N1 pandemic influenza seroepidemiologic studies.^7^, ^8^ Debate has also arisen around the interpretation of seroepidemiologic studies of avian influenza A virus (AIV) infections of humans, given uncertainties about assay performance and antibody kinetics in exposed and unexposed populations.^9^, ^10^, ^11^ In addition, new immunoassays and modifications of well‐established assays are increasingly being used for the detection of influenza virus strain‐specific antibodies.^12^, ^13^, ^14^, ^15^, ^16^, ^17^, ^18^ These issues led to the formation in 2010 of the Consortium for the Standardization of Influenza Seroepidemiology (CONSISE).^19^ CONSISE is comprised of international scientists experienced in conducting seroepidemiologic studies of influenza and other emerging respiratory viruses; two working groups on epidemiology and laboratory matters were formed to provide tools to help standardize protocols and laboratory methods used (see https://consise.tghn.org/about/working-group-projects/). The overarching goal of CONSISE is to improve the quality of data arising from influenza seroepidemiologic studies, harmonize methods used in such studies, and thereby provide better evidence for policy makers that guides rational implementation of intervention and control measures.^19^

Guidelines for the reporting of the design, conduct, and results of research have been an effective tool for improving the quality and interpretability of published data. Examples include the Consolidated Standards of Reporting Trials (CONSORT) and the Strengthening the Reporting of Observational Studies in Epidemiology (STROBE).^20^, ^21^ These guidelines have become, in some instances, widely accepted standards for reporting of research studies, and the expectation that publications should meet these standards has helped to improve the design and conduct of studies. CONSISE has prepared the following statement, *R*eporting *O*f *S*ero‐*E*pidemiologic *S*tudies for *I*nfluenza (ROSES‐I), which distills the experience of the working groups into a set of recommendations on the optimal reporting of influenza seroepidemiologic studies.

### 1.1 Objective

The aim of the CONSISE ROSES‐I statement was to improve the quality and transparency of reporting of seasonal, avian, and pandemic influenza seroepidemiologic studies in order for the validity and generalizability of the results to be better assessed. This statement also aims to improve the design and conduct of influenza seroepidemiologic studies by proposing reporting standards that investigators should consider when designing studies. CONSISE has developed a number of protocols as guides to the design and implementation of seroepidemiologic studies, and these protocols (available at https://consise.tghn.org/articles/available-consise-influenza-protocols/) are a valuable resource that should be consulted in addition to the ROSES‐I statement (Table 1).

**Table 1:** Seroepidemiologic investigation templates^a^ by CONSISE

Available protocol^a^	Primary objectives	Strengths	Weaknesses	Importance of timing of sampling
Pandemic influenza or novel/emerging respiratory viruses				
Prospective longitudinal cohort study of influenza virus infections	Estimate age‐specific incidence rates and cumulative incidence of infections during an influenza epidemic	Provides age‐specific rates, monitors ongoing transmission rates, uses matched serum samples from enrolled participants, can measure the asymptomatic infection rates	Resource intensive, loss to follow up if closed cohort; limited time for initial recruitment and completion of baseline sample collection	Important: baseline before epidemic or as soon as possible. Follow‐up can be anytime after epidemic
Cross‐sectional seroprevalence study of a novel influenza A virus infection prior to and post‐outbreak or post‐epidemic periods	Estimate age‐specific cumulative incidence of infection with a novel influenza A virus in the populationEstimate prevalence of cross‐reactive antibodies to the novel virus among exposed persons and general population	Provides the same age‐related immunity estimates as longitudinal study; less resource‐intensive than longitudinal study; no concern about loss to follow up; select the specific age groups to target	Cannot follow the same participants as longitudinal study; increased risk for bias and interperson variability; may not be matched for location or population makeup; cannot measure asymptomatic infection rates	Important: baseline before epidemic or as soon as possible. Follow‐up can be anytime after epidemic
Household transmission studies of influenza	Estimate household secondary infection risk, and factors associated with variation in the secondary infection riskCharacterize secondary cases including clinical presentation and asymptomatic fractionInvestigate humoral immune response by serology following confirmed influenza virus infection	Provides age‐specific rates, monitors ongoing transmission rates, uses serial serum samples from enrolled participants, can measure the asymptomatic infection rate as well as clinical severity and duration; can measure duration of infectiousness; can measure secondary rate of infection; can test effectiveness of interventions	The most resource‐intensive study design; loss to follow up if closed cohort; need to recruit during widespread circulation in community; challenges in the selection of participating households	Important: baseline before epidemic or as soon as possible. Follow‐up required after every illness episode within the household
Closed settings (e.g., military, child or elderly care centers, prisons) outbreak investigation protocol for influenza or novel respiratory virus	Describes the clinical spectrum of infection including the asymptomatic fractionEstimate overall clinical attack rates (by subgroup and clinical risk group)Describe the correlation between infection, disease, and detection of antibodies by serology	Provides age‐specific rates, monitors ongoing transmission rates, serial serum samples from enrolled participants, can measure the asymptomatic infection rate as well as clinical severity and duration; can measure the duration of infectiousness; can measure the secondary rate of infection; test the effectiveness of interventions	Not as generalizable as it targets specific groups at risk; may give only restricted age group information; may not be totally closed setting (e.g., visitors, outings); length of stay in study setting may not span the whole epidemic period	Important: baseline before epidemic or as soon as possible. Follow‐up required after every illness episode within the setting
Assessment of influenza virus infection in healthcare personnel	Detect evidence of human‐to‐human transmission of a novel influenza A virus within a healthcare setting	Identifies occupational risks of transmission and acquisition of infectious agent; assesses interventions targeting healthcare providers; low rates of loss to follow up	Not as generalizable as it targets only healthcare providers; may not be totally closed setting; may need to be tailored for pathogen‐specific characteristics	Sera can be collected anytime depending upon emergence or prevalence of virus of interest
Seasonal influenza viruses				
Seroepidemiology of human influenza A or B virus infections using residual sera/convenience samples for establishing baseline seroprevalence and/or monitoring trends over time	Estimate population humoral immune status/susceptibility to currently circulating seasonal influenza virus strainsEstimate incidence in previous seasons for different influenza virus strains	Age and demographic factors known in advance; least resource intensive of all protocols; samples are already collected	Population characteristics (age, gender, comorbidities) may be restricted; mostly cross‐sectional; cannot provide individual infection rates@@Interpretation of laboratory results (e.g., seroincidence is challenging)	Sera can be collected anytime
Zoonotic influenza viruses or emerging respiratory viruses				
Investigation of zoonotic influenza A virus infections in humans	Measure age‐specific serologic evidence of infection in relation to zoonotic exposuresIdentify the risk factors for human infection by novel influenza A viruses	Age‐specific infection rates (zoonotic exposure); can identify modifiable risk factors; can quantify the proportion of asymptomatic infections; assesses the potential human‐to‐human transmission; comparative analysis of human and animal influenza A viral strains	Subject to outbreak unpredictability (planning); may be politically charged environment; location may be hard to reach; source of infection could be in a complex and diverse ecosystem; need to coordinate with animal health authorities	Important: 4–6 wk after confirmed outbreak with optional longitudinal follow‐up every 6 wk until outbreak is over

a CONSISE protocols are available here: https://consise.tghn.org/articles/.

The components of the ROSES‐I statement can be used as a checklist to help guide what key information should be included in the results of published seroepidemiologic studies, and can also serve as a guide to the items that need to be considered during study design and implementation. As with other reporting guidelines, this statement is not intended as a required framework that must be followed in content and format. It is also not designed as an instrument for assessing study quality, for which other instruments exist^22^, ^23^.

## 2 Methods

The need for the ROSES‐I statement was agreed at the 4th International CONSISE meeting held on September 3–4, 2013, in Cape Town, South Africa.^24^ The ROSES‐I statement constitutes an expert consensus on the key information that should be considered when reporting the results of seroepidemiologic investigations of influenza and other emerging viruses in order for the validity and generalizability of the results to be assessed by third parties, and to allow comparisons and inferences across study populations. This statement consolidates the recommendations of the CONSISE epidemiology and laboratory working groups into the familiar format of a reporting guideline. It was developed by CONSISE members (PH, BC, JW, OE, KLL, MVK) with input by the CONSISE Steering Committee and other CONSISE members. Because seroepidemiologic studies are a specific kind of observational study, this ROSES‐I statement has built upon the STROBE guidelines in order to avoid confusion and conflicts. As such, the ROSES‐I statement should be seen as an extension of the STROBE guidelines, in the same way that the STrengthening the REporting of Genetic Association Studies (STREGA) guidelines are an extension of STROBE for gene–disease association studies.^25^

### 2.1 Search strategy and selection criteria

References for this review were identified through a search of PubMed conducted on May 15, 2014, and updated in May 2015 using the term (“Seroepidemiologic Studies”[Mesh] AND “Influenza, Human”[Mesh]). The title and abstract of 255 articles published in English were reviewed and information from relevant studies was included in the review. Individual seroepidemiologic studies are referenced only where they demonstrate a principal of relevance to the ROSES‐I reporting standards.

The ROSES‐I standards comprise a checklist of items that should be addressed in addition to the STROBE items, or are suggested refinements of STROBE items, which are specifically applicable to seroepidemiologic studies. Where the existing STROBE item covers the issue in full and no specific addition or refinement is required for seroepidemiologic studies, the STROBE item is given in tables, but there is no corresponding ROSES‐I item.

## 3 Results

### 3.1 ROSES‐I standards

#### 3.1.1 Title and abstract

To facilitate the clear identification of studies that quantitatively measure antibodies concentration in members of a defined population in order to make inferences about exposure of that population to emerging respiratory viruses, transmission, and severity, one of the terms “seroepidemiologic,” “seroepidemiology,” “seroprevalence,” or “seroincidence” should be used in the title and/or abstract of the study, and the MeSH term “Seroepidemiologic Studies” should be used as a keyword [ROSES‐I 1.1] (Table 2).

**Table 2:** Components of the ROSES‐I statement for standardization of the reporting of seroepidemiologic studies^a^

Item	Item number	STROBE items	ROSES‐I items
Title and abstract	1	Indicate the study's design with a commonly used term in the title or the abstract Provide in the abstract an informative and balanced summary of what was done and what was found	ROSES‐I 1.1: The term “seroepidemiologic,” “seroepidemiology,” “seroprevalence,” or “seroincidence” should be applied to the study in the title or abstract, and the medical subject heading “Seroepidemiologic Studies” be used when the report is of a population‐based serological survey
Introduction	2	Explain the scientific background and rationale for the investigation being reported	ROSES‐I 2.1: State what is known about the kinetics of antibody rise, decay, and persistence following infection for the particular virus being studied and the justification for threshold antibody titers or changes in titers used to define evidence of infection
			ROSES‐I 2.2: State what is known about the sensitivity and specificity of the antibody detection assay being used
	3	State specific objectives, including any prespecified hypotheses	ROSES‐I 3.1: State the specific measure of occurrence that is being estimated, for example, point seroprevalence, cumulative incidence of infection, secondary infection risk
Epidemiologic methods			
Study design	4	Present key elements of study design early in the paper	ROSES‐I 4.1: State which specific seroepidemiologic study design was chosen and why (see Table 1)
Setting	5	Describe the setting, locations, and relevant dates, including periods of recruitment, exposure, follow‐up, methods for sampling, and data collection	ROSES‐I 5.1: Describe the timing of the biological sampling in relation to the disease epidemiology in the study population (the beginning, peak, and end of virus transmission)
			ROSES‐I 5.2: Where known, describe the timing of biological sampling in individuals in relation to disease onset and to exposures of interest
			ROSES‐I 5.3: State the interval between sequential biological samples (serial cross‐sectional or longitudinal studies), or specify whether only a single sample was collected (cross‐sectional study)
Participants	6	Cohort study—Give the eligibility criteria, and the sources and methods of selection of participants. Describe methods of follow‐upCase–control study—Give the eligibility criteria, and the sources and methods of case ascertainment and control selection. Give the rationale for the choice of cases and controlsCross‐sectional study—Give the eligibility criteria, and the sources and methods of selection of participants Cohort study—For matched studies, give matching criteria and the number of exposed and unexposedCase–control study—For matched studies, give matching criteria and the number of controls per case	ROSES‐I 6.1: For case‐ascertained transmission studies, describe the method of case ascertainment and criteria for defining a “case”
			ROSES‐I 6.2: For household‐ or institution‐based transmission studies, describe the definition of a household or the institution
			ROSES‐I 6.3: For outbreak investigations involving serologic sampling, describe the setting in which the cases were identified, for example, village/residential setting, occupational workplace
			ROSES‐I 6.4: To aid the interpretation of seroepidemiologic studies of novel influenza A virus subtypes, the results from exposed populations should be compared with the results from unexposed populations. Efforts to validate the assay in virologically confirmed cases should be reported
Variables	7	Clearly define all outcomes, exposures, predictors, potential risk factors, and effect modifiers. Give diagnostic criteria, if applicable	ROSES‐I 7.1 The median age and range for each exposure group should be reported
			ROSES‐I 7.2: Describe the potential for immunization (specify vaccine and timing of vaccination in relationship to collection of serum), if applicable, to affect the outcome measures
			ROSES‐I 7.3: Describe any known or potential immunological cross‐reactivity that may bias the outcome measures
			ROSES‐I 7.4: Describe illness definitions and methods for ascertaining the presence or absence of clinical illness in subjects
Data sources/measurement biases	8^a^	For each variable of interest, give sources of data and details of methods of assessment (measurement). Describe comparability of assessment methods if there is more than one group	ROSES‐I 8.1: If relevant, describe measures taken to identify and record immunization history
Bias	9	Describe any efforts to address the potential sources of bias	ROSES‐I 9.1: If relevant, describe efforts to control for the potential effect of immunization on estimates of outcomes
Study size	10	Explain how the study size was arrived at	ROSES‐I 10.1: Describe the baseline estimated seroprevalence at given antibody titers or incidence of infection and cite published literature to support these estimates
Quantitative variables	11	Explain how quantitative variables were handled in the analyses. If applicable, describe which groupings were chosen, and why	ROSES‐I 11.1: Describe the serological assay's limit of detection and how this limit is defined or calculated. Describe how samples with a result below or on the borderline of the limit were handled in the analysis
			ROSES‐I 11.2: Describe and justify the titer or other result used to define “seropositivity,” or the antibody titer change or change in other assay result used to define “seroconversion.” Avoid the term “seroconversion” unless referring to change from undetectable to detectable antibody level. Otherwise report the fold‐rise in titer. Avoid the term “infection” but report “seroprevalence at a titer of ….”
			ROSES‐I 11.3: If statements or inferences are made about protection from infection, describe what is known about the correlation between the assay results and protection from infection and illness
Statistical methods	12	Describe all statistical methods, including those used to control for confounding Describe any methods used to examine subgroups and interactions Explain how missing data were addressed Cohort study—If applicable, explain how loss to follow up was addressedCase–control study—If applicable, explain how matching of cases and controls was addressedCross‐sectional study—If applicable, describe analytical methods taking account of sampling strategy Describe any sensitivity analyses	ROSES‐I 12.1: if relevant, state how the non‐independence of data was managed
			ROSES‐I 12.2: if relevant, report methods used to account for the probability of seropositivity or seroconversion if infected, and to account for decay in antibody titers over time
Laboratory methods	12a		
Sample type and handling			ROSES‐I 12a.1: Describe the sample type—serum or plasma. If plasma is used, specify the anticoagulant used (heparin, sodium citrate, EDTA, etc.)
			ROSES‐I 12a.2: Describe the specimen storage conditions (4°C, −20 °C, −80 °C). If frozen prior to the analysis, describe the time to freezing and the number of freeze/thaw cycles prior to testing
Serological assays			ROSES‐I 12a.3: Specify the assay type (e.g., hemagglutination inhibition; virus neutralization/microneutralization; ELISA; other) and methods used to determine the endpoint titer
			ROSES‐I 12a.4: Reference a previously published, CONSISE consensus serologic assay or WHO protocol if used, and any modifications of the protocol. If a previously published protocol is not used, provide full details in supplementary materials
			ROSES‐I 12a.5: State what is known about the determinants of the variability of the antibody detection assay being used
			ROSES‐I 12a.6: Specify the antigen(s) used in the assay, including virus strain name, subtype, lineage or clade, with standardized nomenclature and reference; specify whether live virus or inactivated virus was used (where applicable)
			ROSES‐I 12a.7: Report if antigen(s) from potentially cross‐reactive pathogens/strains were used in order to identify cross‐reactivity, and specify which antigen was used, including virus name, subtype, strain, lineage and clade, with standardized nomenclature and reference
			ROSES‐I 12a.8: If red blood cells were used for a hemagglutinin inhibition assay, specify the animal species from which they were obtained and concentration (v/v) used
			ROSES‐I 12a.9: Describe positive and negative controls used
			ROSES‐I 12a.10: Describe starting and end dilutions
			ROSES‐I 12a.11: Specify laboratory biosafety conditions
			ROSES‐I 12a.12: Specify whether replication was performed, and if so, the acceptable replication parameters
			ROSES‐I 12a.13: Specify whether a confirmatory assay was performed and all specifics of this assay, at the same level of detail
			ROSES‐I 12a.14: Specify international standards used, if appropriate
Results			
Participants	13^a^	Report the numbers of individuals at each stage of the study—the numbers potentially eligible, examined for eligibility, confirmed eligible, included in the study, completing follow‐up, and analyzed Give reasons for non‐participation at each stage Consider use of a flow diagram	See STROBE item
Descriptive data	14^a^	Give characteristics of study participants (e.g., demographic, clinical, social) and information on exposures and potential risk factors Indicate the number of participants with missing data for each variable of interest Cohort study—summarize follow‐up time (e.g., average and total amount)	See STROBE item
Outcome data	15^a^	Cohort study—report the numbers of outcome events or summary measures over timeCase–control study—report the numbers in each exposure category, or summary measures of exposureCross‐sectional study—report the numbers of outcome events or summary measures	See STROBE item
Main results	16	Give unadjusted estimates and, if applicable, risk factor‐adjusted estimates and their precision (e.g., 95% confidence interval). Make clear which risk factors were adjusted for and why they were included Report category boundaries when continuous variables were categorized If relevant, consider translating estimates of relative risk into absolute risk for a meaningful time period	ROSES‐I 16.1: Report unadjusted estimates of distribution of titers by age group
			ROSES‐I 16.2: Report methods to standardize the results from the study sample to the target population
Other analyses	17	Report other analyses performed—analyses of subgroups and interactions, and sensitivity analyses	See STROBE item
Discussion			
Key results	18	Summarize key results with reference to study objectives	See STROBE item
Limitations	19	Discuss limitations of the study, taking into account sources of potential bias or imprecision. Discuss both direction and magnitude of any potential bias	ROSES‐I 19.1 Discuss limitations and strengths of the study with reference to Table 1
Interpretation	20	Give a cautious overall interpretation of results considering objectives, limitations, multiplicity of analyses, results from similar studies, and other relevant evidence	ROSES‐I 20.1: Discuss the interpretation of the results in the context of known or potential cross‐reactivity
Generalizability	21	Discuss the generalizability (external validity) of the study results	See STROBE item
Other information			
Funding	22	Give the source of funding and the role of the funders for the present study and, if applicable, for the original study on which the present article is based	ROSES‐I 22.1: Specify if institutional review board approval was received; if not, specify reason (e.g., public health outbreak response/non‐research designation)

a Give such information separately for cases and controls in case–control studies and, if applicable, for exposed and unexposed groups in cohort and cross‐sectional studies.

#### 3.1.2 Introduction

The validity of inferences about virus infection risks based on serology is dependent upon knowledge of the kinetics of antibody responses following virus infection and of the performance of the antibody detection assay. The introduction should provide background information justifying the choice of antibody detection assay and thresholds for specific antibody titer or changes in antibody titer, which may be indicative of prior or recent infection [ROSES‐I 2.1 and 2.2]. Depending on their design, seroepidemiologic studies can provide data on a variety of measures of the frequency of an outcome in the study population. For example, cross‐sectional seroepidemiologic studies provide estimates of the point prevalence of different titers of antibodies, which are used as markers of prior infection (or vaccination) and may indicate the current level of antibody‐mediated protection against infection with antigenically similar viruses if protective thresholds or correlates of protection have been established. The wording “seroprevalence at an antibody titer of ….” is preferred to “infection,” because antibody titers are dynamic, initially rising and then generally declining during variable periods after infection, and therefore, different assumptions underlie inferences about prior infection from antibody concentrations. Serological testing of paired serum specimens provides estimates of the cumulative incidence of virus infection (or incidence proportion). Serial cross‐sectional or prospective longitudinal cohort studies can be used to estimate cumulative incidence of virus infection.^4^ Serology can also be used in studies of populations in specific settings, such as households, healthcare settings, or other confined settings (such as military units, child/elderly care centers or prisons), to estimate the risk of secondary virus infections or to estimate the seroprevalence of antibodies in specific populations. The specific measure of occurrence that is being estimated (e.g., point seroprevalence, cumulative incidence, secondary infection risk) should be described in the introduction [ROSES‐I 3.1].

#### 3.1.3 Epidemiological methods

##### *Study design and setting*

A number of different seroepidemiologic study designs can be used to estimate various measures of virus infection risk, and different designs have different strengths and weaknesses depending on the objectives of the investigation (Table 1). The methods section should begin by describing the study design, the study population, sampling procedures (e.g., random or convenience), the source of serum or plasma that was analyzed (e.g., frozen stored vs recently collected and tested), the rationale for choosing the study design, and the generalizability beyond the study population [ROSES‐I 4.1]. Inferences about the probability of prior or recent virus infection based on the titer of antibody in a single serum sample, or changes in the titer of antibody in sequential serum samples, are based upon assumptions about the kinetics of antibody titers following virus infection. In order for readers to assess or test the validity of the assumed relationship, it is important that details are provided of the actual or likely period of exposure(s) to the circulating virus strain and infection risk in relation to the time point that the serum samples are taken [ROSES‐I 5.1‐5.3]. For example, an overly short (<14 days) or overly long (>6 months) interval between exposure(s) and serum sampling will increase the probability of uninterpretable results because antibody titers may not yet have reached a maximum titer, or may have decayed below the seroprevalence or seroconversion titer detection thresholds set to define infection retrospectively.

##### *Participants*

For studies where close contacts of confirmed influenza cases (e.g., household contacts of confirmed cases; Table 1: household transmission studies) are monitored for seroconversion in order to estimate the incidence of secondary infections, in addition to the STROBE criteria for reporting study subject details, the method and criteria for identifying index case patients should be reported because the probability of onward transmission may vary according to the characteristics of the index case patients (Table 2). Thus, comparison of the results of different case‐ascertained virus transmission studies requires this information [ROSES‐I 6.1]. For example, subjects with a clinical illness that is severe enough to seek medical care, or those with preexisting comorbid conditions, immunosuppression, or with poorly developed immune systems (e.g., young infants), may be more infectious than mild case patients detected through active follow‐up of case contacts. Similarly, the virus load in clinical specimens, and therefore infectivity, may differ among subjects with infections detected by virus culture, molecular diagnostic techniques, or rapid antigen detection point‐of‐care (POC) diagnostic tests. In addition, the duration, intensity, and route of exposure during the index case patient's illness will influence the infectiousness of the index case patient to close contacts. For studies of virus infection transmission risk in households or closed settings, or for outbreak investigations, the definition of the setting being studied (e.g., how a household was defined) should be described in order to permit comparison with other similar studies [ROSES‐I 6.2‐6.3]. The results of seroepidemiologic studies among humans of antibodies to novel avian influenza viruses (AIVs) have sometimes been challenging to interpret, because it is difficult to distinguish the detection of low titers of virus‐specific antibodies from the detection of low titers of cross‐reactive antibodies. As such, seroepidemiologic surveys of novel AIVs should ideally compare the seroprevalence in exposed populations to the seroprevalence in populations who have likely not been exposed to the animal reservoir or to sick human case patients, and should report the efforts to validate the assay in virologically confirmed cases [ROSES‐I 6.4].^11^, ^15^, ^26^

##### *Variables, data sources, and bias*

Age is an important determinant of serologic responses to virus infection, and therefore, it is critical that the median and range of the age of participants, overall and by subgroup (study subjects and control population; the control group should have a similar median age and age range to the study group.), should be reported (ROSES‐I 7.1). For influenza, antibody may be present as a result of immunization. It is therefore essential that where a vaccine exists, details are provided of the vaccine(s) used in the study population, when vaccination was administered in relation to the timing of sample collection during the investigation [ROSES‐I 7.2], the methods used to identify and record vaccination history [ROSES‐I 8.1], as well as statistical methods used to control for the potential effect of immunization [ROSES‐I 9.1]. When no data are available on vaccination in the study population or the general population from which the study participants were chosen, this should be stated clearly along with any anecdotal information as to the presence or absence of vaccination during the period of study. When pathogens share similar antigenic epitopes, antibody assays may detect cross‐reactivity to more than one pathogen. For example, antibodies that cross‐reacted with the influenza A(H1N1)pdm09 virus were detected in serum samples collected prior to the emergence of the 2009 H1N1 pandemic virus. These cross‐reactive antibodies were typically found in older people who were exposed to earlier influenza A(H1N1) viruses, particularly the 1918 H1N1 virus.^27^ Therefore, any known or potential immunological cross‐reactivity and efforts to detect and control for cross‐reactivity should also be reported [ROSES‐I 7.3; 12a.7]. Influenza virus infection can result in a wide range of clinical outcomes, from asymptomatic infection to a rapidly progressive fatal illness. Different definitions of clinical illness and different methods of ascertaining the presence or absence of clinical illness will have variable sensitivity and specificity for detecting an influenza‐associated illness. The definitions and methods used to identify the clinical outcomes should be described [ROSES‐I 7.4].

##### *Study subjects and sample size*

In order to permit the generalizability of the study results, it is preferable that the study population be as close as possible to the general population under study. However, this is not always feasible, and for example, many serologic studies detecting antibodies to A(H1N1)pdm09 virus infection were conducted using residual sera from blood donors^4^, ^28^ or hospitalized patients,^29^ who may not be representative of the broader populations in those locations. The potential to introduce selection bias into the study should be addressed in the discussion. Any method used to infer cumulative incidence of infection among the population based on results from the study sample, for example, weighting or standardization, should be reported in sufficient detail to permit reproducibility (ROSES‐I 16.2).

In addition, the confidence in the results and conclusions of any seroepidemiologic study depends, among other things, on whether the planned study sample size was sufficient to provide estimates of prevalence or incidence of infection with sufficient precision and certainty.^30^ To assess whether the planned sample size was adequate, the assumed baseline prevalence of given antibody titers or the baseline cumulative incidence of a given change in antibody titer should be reported [ROSES‐I 10.1]. Differences between the planned and actual sample size should also be reported, and any differences explained.

##### *Quantitative variables*

For seroepidemiologic studies, it is particularly important that full details and conditions of assay methods to detect antibodies are reported including the upper and lower limits (antibody dilution range) of assay detection. In addition, variable conditions of the assays should be described. The minimum detection level of the assay, and how this limit is defined or calculated, should be reported, as should the handling in the analysis of samples with a result below or on the borderline of the minimum detection level [ROSES‐I 11.1]. A result below the minimum detection level of the assay is consistent with an antibody titer of anywhere between zero and just below the limit of detection. How these titers are analyzed and interpreted can affect the results, especially if they constitute a large proportion of the results. A common convention, which is acceptable, is to consider a result below the limit of detection as a serial step below that limit; that is, if the starting antibody dilution is 10, then a value of <10 can be reported as a five for the purposes of data analysis rather than a zero or a “not detected.” Similarly, antibody titer thresholds to define a seropositive result are critically important yet are, in some studies, fairly arbitrary. Thresholds that are designed with high specificity for diagnostic purposes in the cases of clinical illness may not provide reliable estimates of virus infection rates at the population level, if specificity has been optimized at the expense of sensitivity.^31^ Therefore, the criterion for defining a positive result (as either an indication of prior infection or to infer protection) and the justification for using this criterion in the particular study setting must be reported [ROSES‐I 11.2]. Immunological correlates of protection from influenza virus infection and illness are difficult to establish and the relationship between antibody titer and protection is not binary (completely protected above a titer threshold and completely unprotected below the threshold).^32^, ^33^ Therefore, if results are used to make inferences about the proportion of a population or population subgroup that are “protected,” it is important that this is justified by reference to what is known about the correlation between antibody titers measured by the specific assay and protection from infection or illness [ROSES‐I 11.3].

##### *Statistical methods and presentation of results*

In cross‐sectional studies, seroprevalence can be estimated by the proportion of specimens with antibody titers at or above a specific threshold, with 95% confidence intervals typically obtained using the binomial formula or the normal approximation to the binomial. If a number of additional assumptions are met, including that seroprevalence before an epidemic is very low, and almost all infected individuals have rises in convalescent antibody titers above the chosen threshold, the post‐epidemic seroprevalence can provide an approximate estimate of the cumulative incidence of infection.^34^ Note that seroprevalence is a proportion and not a rate.

In studies with paired sera, the cumulative incidence of virus infection can be estimated by the proportion of persons with a rise in antibody titer, traditionally a fourfold or greater rise.^31^ In most studies, 95% confidence intervals are typically estimated using the binomial formula or the normal approximation to the binomial, implicitly assuming that each person can experience no more than one virus infection during the period considered. It is noteworthy to point out also that cumulative incidence of virus infection is sometimes referred to as an “attack rate,” although a proportion of infections may be asymptomatic (and therefore not “attacks”), and the quantity measured is a proportion and not a rate. The term “cumulative incidence of infection” should therefore be preferred to “attack rate” in the context of serological studies.

In either case, the methods used to account for the probability of seropositivity or seroconversion if infected, and any method used to account for decay in antibody titer over time, should be reported (ROSES‐I 12.2). To increase transparency of cumulative incidence of infection estimates, it is often helpful to report unadjusted estimates of the distribution of antibody titers by age group (ROSES‐I 16.1).

In some studies, particularly those with more complex designs in terms of timing of serologic measurements, improved estimates of the seroprevalence at a certain point in time, or the cumulative incidence of infection over a specified time period, may be obtained by fitting observed data to a mechanistic model of transmission dynamics.^4^, ^35^ This can account for non‐independence in the data (ROSES‐I 12.1).

#### 3.1.4 Laboratory methods

##### *Sample type and handling*

Although serum samples are more commonly used for serologic studies, convenience sampling may only enable access to plasma. The use of anticoagulants to separate plasma has been shown to reduce the antibody titer to some influenza viruses.^36^ Thus, defining the sample type and anticoagulant, if used, is necessary (ROSES‐I 12a.1). The storage conditions, duration, and subsequent treatment of samples (e.g., temperature and the number of freeze/thaw cycles) are important to report, if known, as repeated freeze/thaw cycles may also reduce the antibody titer (ROSES‐I 12a.2).

##### *Serologic assays*

Where possible, standard serologic assays [e.g., hemagglutination inhibition (HAI) assay to detect HAI antibodies or microneutralization (MN) assay to detect neutralizing antibodies for influenza viruses] should be used.^37^All specimen preparation and assay protocols should be provided, either referenced to a published protocol, with any changes specified, or as detailed methodology for novel serologic assays (ROSES‐I 12a.3, 4). Any parameters that may induce variability of the antibody detection assay being used should be stated (ROSES‐I 12a.5). It is necessary to report all details of the antigen used, including the virus name, subtype, strain, lineage or clade as well as preparation type (e.g., live virus, inactivated virus, recombinant protein). This antigen should be antigenically equivalent to the specific virus strain to which the study population was exposed. To enable the comparison between laboratories and also aid in the development of the specific serologic assay in other laboratories, all detection parameters (red blood cell species and concentration (as percent v/v), ELISA substrate, starting and end dilutions, etc.), the number of replicates performed and controls, as well as biosafety requirements, should be reported (ROSES‐I 12a.6, 12a.8‐12a.10, 12a.11). The definition and method of endpoint titer calculation should be stated. Interpretation of the data is reliant on appropriate description of the limitations of each serologic assay (ROSES‐I 12a.5) and the reproducibility of the assay (ROSES‐I 12a.12). Performance of additional serological assays to confirm or calibrate results, if appropriate, should be included. Specifically, as the WHO recommendation for A(H5N1) viruses recommends the use of confirmatory serologic assays upon the detection of single serum positive by MN assay, any confirmatory assays used and the criteria for positivity also need to be described in the same details as above (ROSES‐I 12a.13).^38^, ^39^ Inclusion of available international standards^40^, ^41^ facilitates the comparability of serological data. Inclusion of the actual titers obtained from the international standards and indication whether the data are reported as raw values or international standard‐adjusted values should therefore be described (ROSES‐I 12a.14).

##### *Results*

Unadjusted estimates of titers by age group should be reported (ROSES‐I 16.1) and where the results from the study sample have been standardized to a general or target population, the method of standardization should be reported (ROSES‐I 16.2).

##### *Discussion*

Different seroepidemiologic study designs have different strengths and weaknesses, which have been reviewed and summarized by the CONSISE group (Table 1). The limitations and strengths of the study design being reported should be covered in the discussion (ROSES‐I 19.1). Table 1 may be a useful reference for discussing limitations and strengths. Because the interpretation of seroepidemiologic studies is critically dependent on the specificity of the assay, in addition to the STROBE item, specific attention should be given to the interpretation of the results in the context of known or potential cross‐reactivity [ROSES_I 20.1].

## 4 Conclusions

The direct comparability of influenza seroepidemiologic studies is currently limited by a lack of standardization across such studies.^8^ The ROSES‐I statement aims to improve the quality and transparency of the reporting of such studies so that such studies can be better assessed and understood. Here, we have outlined which methodological details―study design, study population, epidemiologic data collection, specimen collection and handling methods, laboratory methods, justification of criteria for seropositivity, reporting of results, limitations and biases, and interpretation―should be included when reporting the findings of seroepidemiologic studies.

Our aim is for the ROSES‐I, like other standards of reporting (e.g., CONSORT, STROBE), to be developed and accepted as the standard for reporting of influenza seroepidemiologic studies. When novel influenza A viruses emerge, a rapid and robust evaluation of the implications of seroepidemiologic studies is critical in order to fully assess the population health risks and the need for mitigation measures. Without the ROSES‐I‐recommended information for reporting, our ability to interpret seroepidemiologic studies of novel influenza A viruses will be limited.

This is the first version of the ROSES‐I checklist, and we hope this will be refined with use and feedback. The approach used by CONSISE for influenza seroepidemiologic studies and the items outlined in this statement are likely to be applicable or adaptable to other emerging respiratory viruses, such as Middle East respiratory syndrome coronavirus (MERS‐CoV); however for simplicity, the ROSES‐I statement described here is focused on influenza viruses. For this reason, we have entitled this statement ROSES‐I, to allow for extension of the acronym ROSES to other such pathogens.

## Appendix 1

### CONSISE Steering Committee Members

The members of the steering committee of CONSISE include Eeva Broberg and Angus Nicoll from ECDC, John Wood (retired) and Othmar Engelhardt of NIBSC UK, Wenqing Zhang from WHO; Anthony Mounts, Jackie Katz, and Tim Uyeki from US CDC; Karen Laurie from the WHO Collaborating Centre for Reference and Research on Influenza, Melbourne, Australia; Maria Van Kerkhove from the Center for Global Health, Institut Pasteur; Steven Riley from the MRC Centre for Outbreak Analysis and Modelling, Imperial College London, UK; Benjamin Cowling and Malik Peiris from the School of Public Health, The University of Hong Kong, Hong Kong; Katja Hoeschler and Richard Pebody from Public Health England, London, UK; Peter Horby from the Oxford University Clinical Research Unit in Hanoi, Vietnam; Barbara Raymond from PHAC; Marianne van der Sande from RIVM and Olav Hungnes from the Norwegian Institute of Public Health, Oslo, Norway.
